# Supplementary material for: Reconstructing cell cycle pseudo time-series via single-cell transcriptome data
Source: Nat Commun. 2017 Jun 19;8:22. doi: 10.1038/s41467-017-00039-z (PMC5476636; doi:10.1038/s41467-017-00039-z)
Supplement: Supplementary file 1 — Supplementary Information [file 41467_2017_39_MOESM1_ESM.pdf]

File name: Supplementary Information
Description: Supplementary figures, supplementary tables, supplementary notes, supplementary methods and supplementary references.

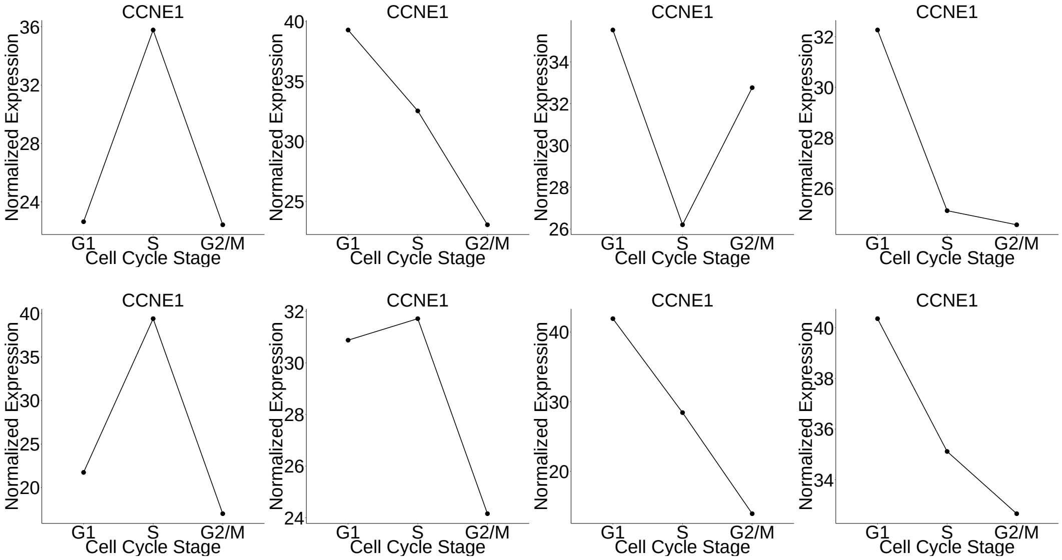

Supplementary Figure 1 | High stochasticity of single-cell gene expression means, as demonstrated by relative expression levels of gene *Ccne1* using the mESC-SMARTer data. For every panel, 20 sample cells were randomly selected for each of the three stages, followed by plotting the mean expression levels at each stage. According to existing records, *Ccne1* has its peak of relative expression at S stage. However, the panels exhibited high stochasticity for the peak expression stage.

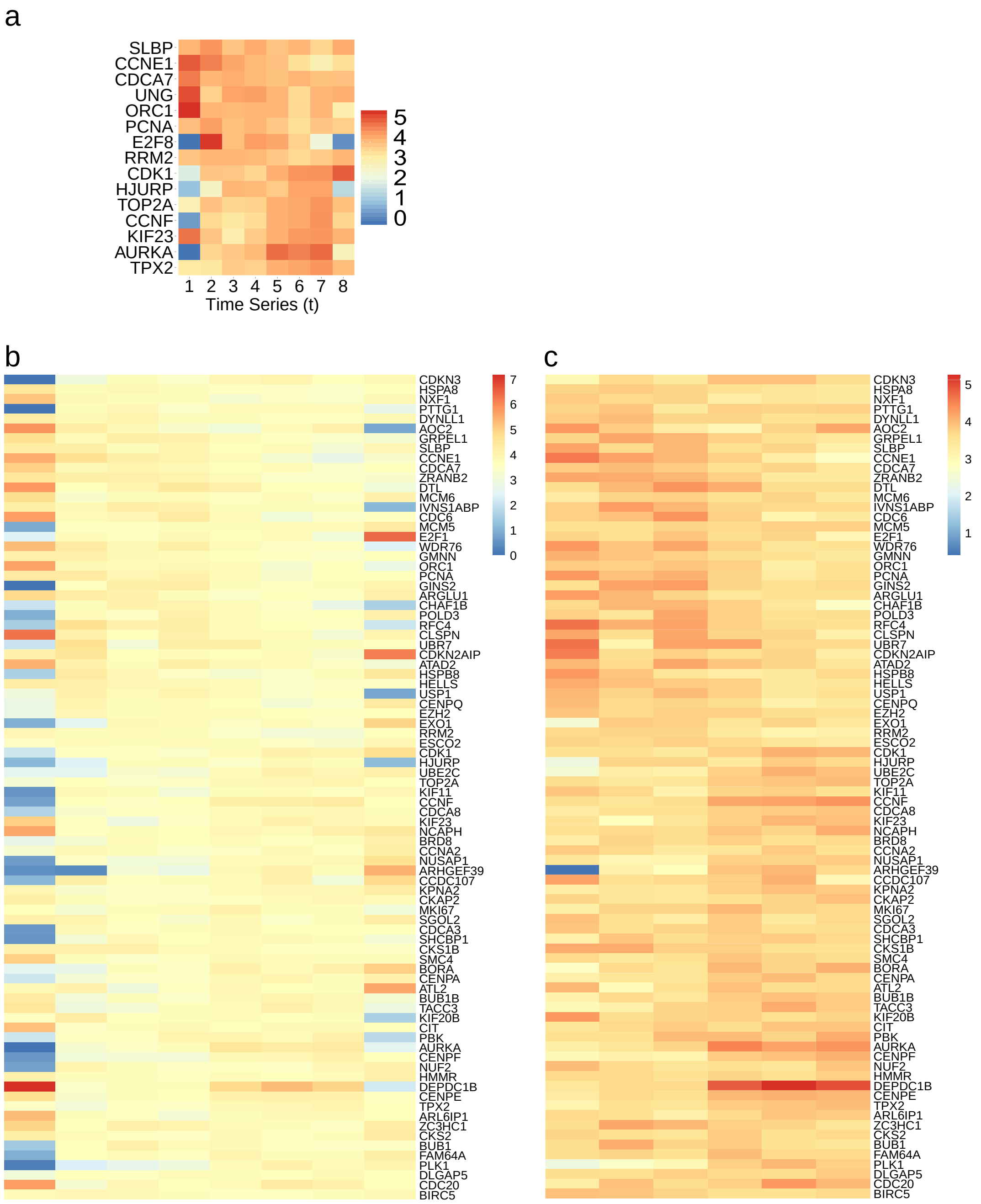

Supplementary Figure 2 | High-belief cell cycle genes used to validate the illustrated cell cycle time-series in the main text. The time-series was generated for the mESC-SMARTer data with predefined cluster number $K = 8$. The gene expression heatmaps were normalized twice by DESeq first vertically and then horizontally for better visualization. The expression profile confirmed the correctness of the time-series for the eight clusters. (**a**) Normalized gene expression levels of high-confidence cell cycle marker genes (G1 peak: *Slbp*, *Ccne1*, *Cdca7*, *Ung*; G1/S peak: *Orc1*, *Pcna*; S peak: *E2f8*, *Rrm2*; G2 peak: *Cdk1*, *Hjurp*, *Top2a*, *Cdnf*, *Kif23*; M peak: *Aurka*, *Tpx2*). (**b**) Normalized expression matrix of top 20 Cyclebase genes for each of the six cell cycle stages (Supplementary Table 5): 'G1'-, 'G1/S'-, 'S'-, 'G2'-, 'G2/M'-, and 'M'-, arranged in the physiological order (from 'G1' to 'M') from left to right. (**c**) Same as (b), but with the two small clusters removed (the first and last clusters).

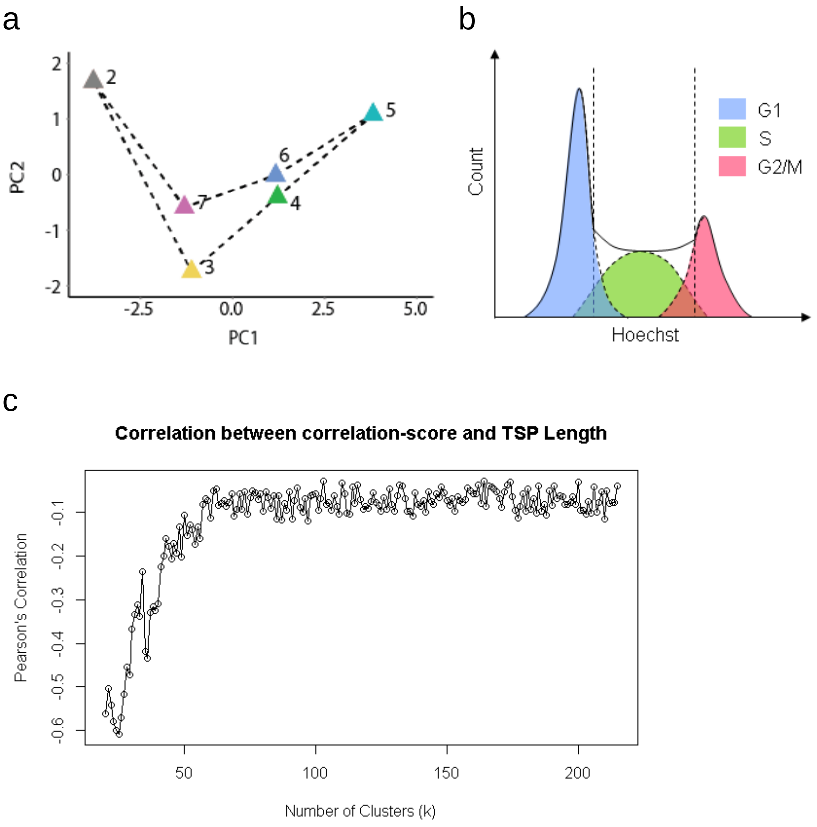

Supplementary Figure 3 | Other properties of the mESC-SMARTer data. (**a**) The 2-dimensional PCA plot for another time-series of the clusters, which was generated by their within-cluster covariance matrices (Supplementary Methods). 'Cluster 1' and 'Cluster 8' were removed before because they contained too few samples to compute an eligible covariance matrix. (**b**) Explanation for incorrect labels by Hoechst staining. The colored regions indicate distributions of G1, S and G2/M cells over Hoechst staining. The two dashed lines represent commonly used thresholds to separate cells to cell cycle stages. Evidently, overlaps between these distributions can cause incorrect labels. (**c**) The Pearson scores between correlation-scores and the TSP distances for different cluster number k using the mESC-SMARTer data. The cluster number ranges from 20 to 215. For each k, we computed a Pearson correlation over 1000 pairs of correlation-scores and TSP distances (by the arbitrary insertion algorithm). The Pearson scores are around -0.6 when the cluster numbers are small, and when the cluster numbers increase, the scores approach -0.07. This shows that the cell cycle can be modeled as a TSP problem.

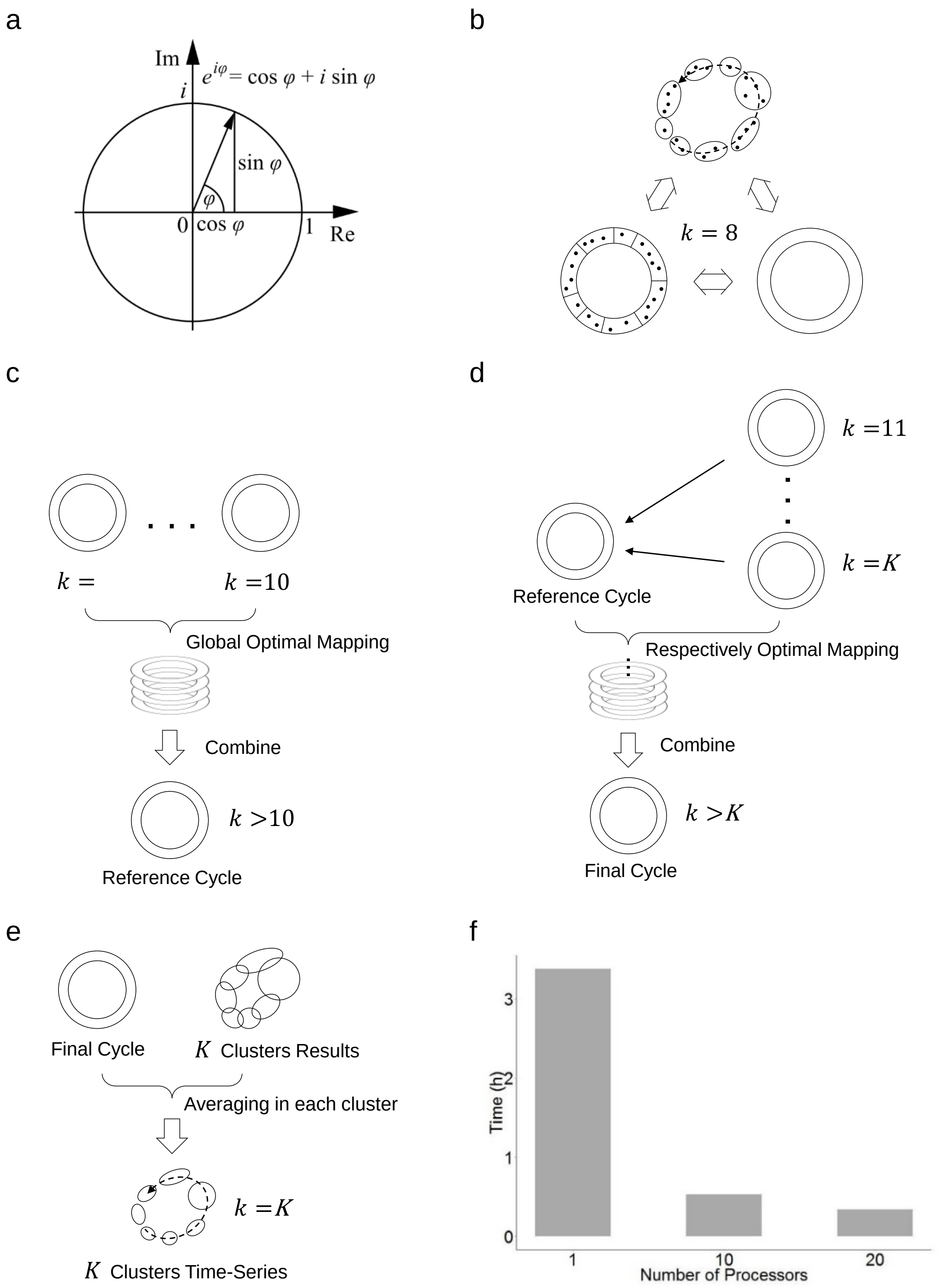

Supplementary Figure 4 | Diagrams to display the procedures of consensus-TSP algorithm. (**a**) The panel exhibits the congruence of three graphic symbols which signify clusters time-series. (**b**) The process of generating a reference cycle. (**c**) The process of generating the final cycle of the cells. (**d**) The process of transforming the final cycle into a K clusters time-series. Running time (hours) of reCAT on the mESC-SMARTer dataset using 1, 10 and 20 cores of a computer with two CPUs (Intel(R) Xeon(R) CPU E5-2680 v2 @ 2.80GHz, and 512GB RAM), each with 10 cores. The script was written in R.

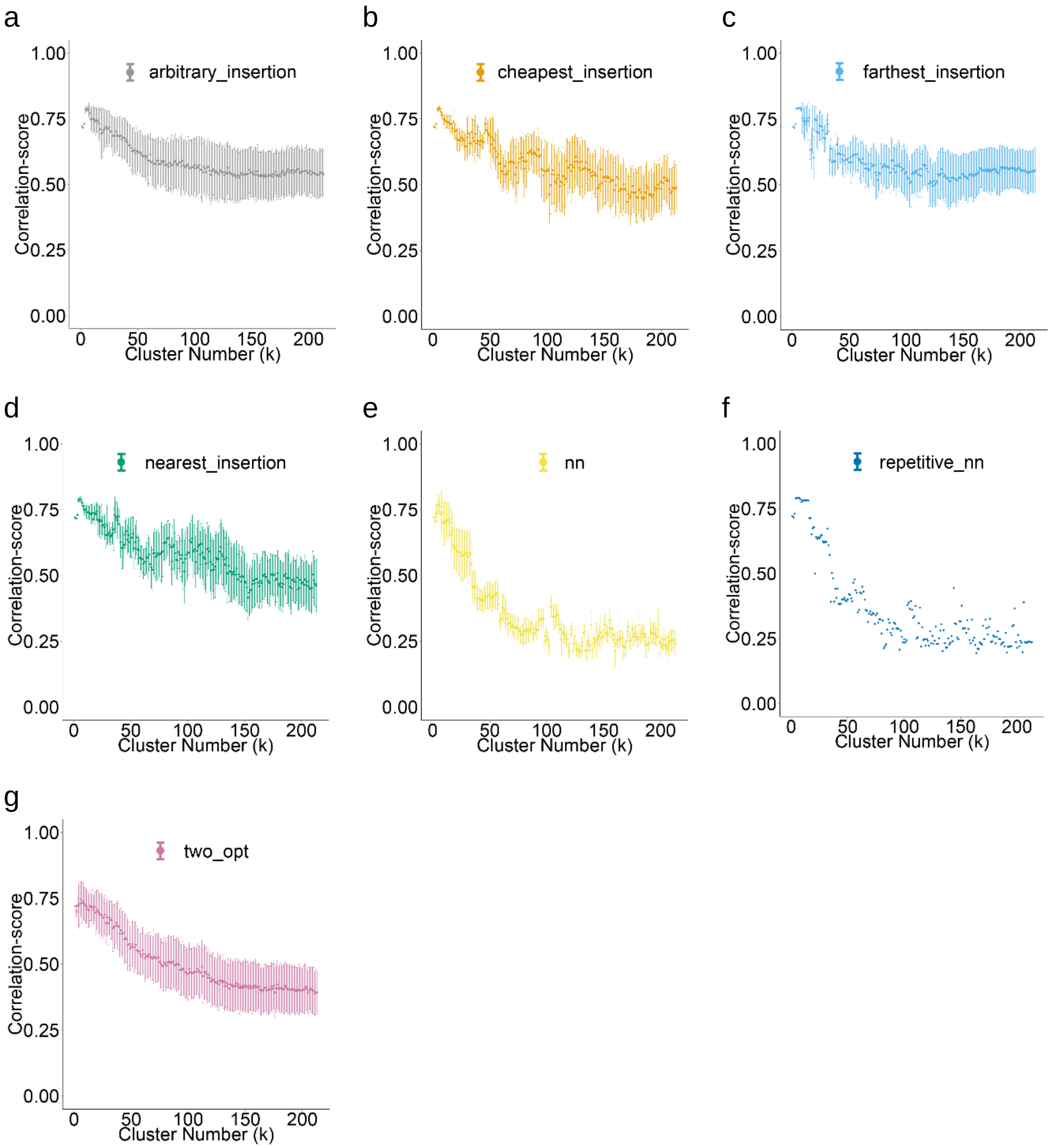

Supplementary Figure 5 | Comparisons of seven algorithms for the TSP problem using the mESC-SMARTer dataset. A half width of the error bars stands for the standard deviation. (**a**) "arbitrary insertion", (**b**) "cheapest insertion", (**c**) "farthest insertion", (**d**) "nearest insertion", (**e**) "nearest neighbor ", (**f**) "repetitive nearest neighbor" and (**g**) "two-optimal". The solutions were assessed by the correlation-score, and the arbitrary insertion and farthest insertion algorithms outperformed the others.

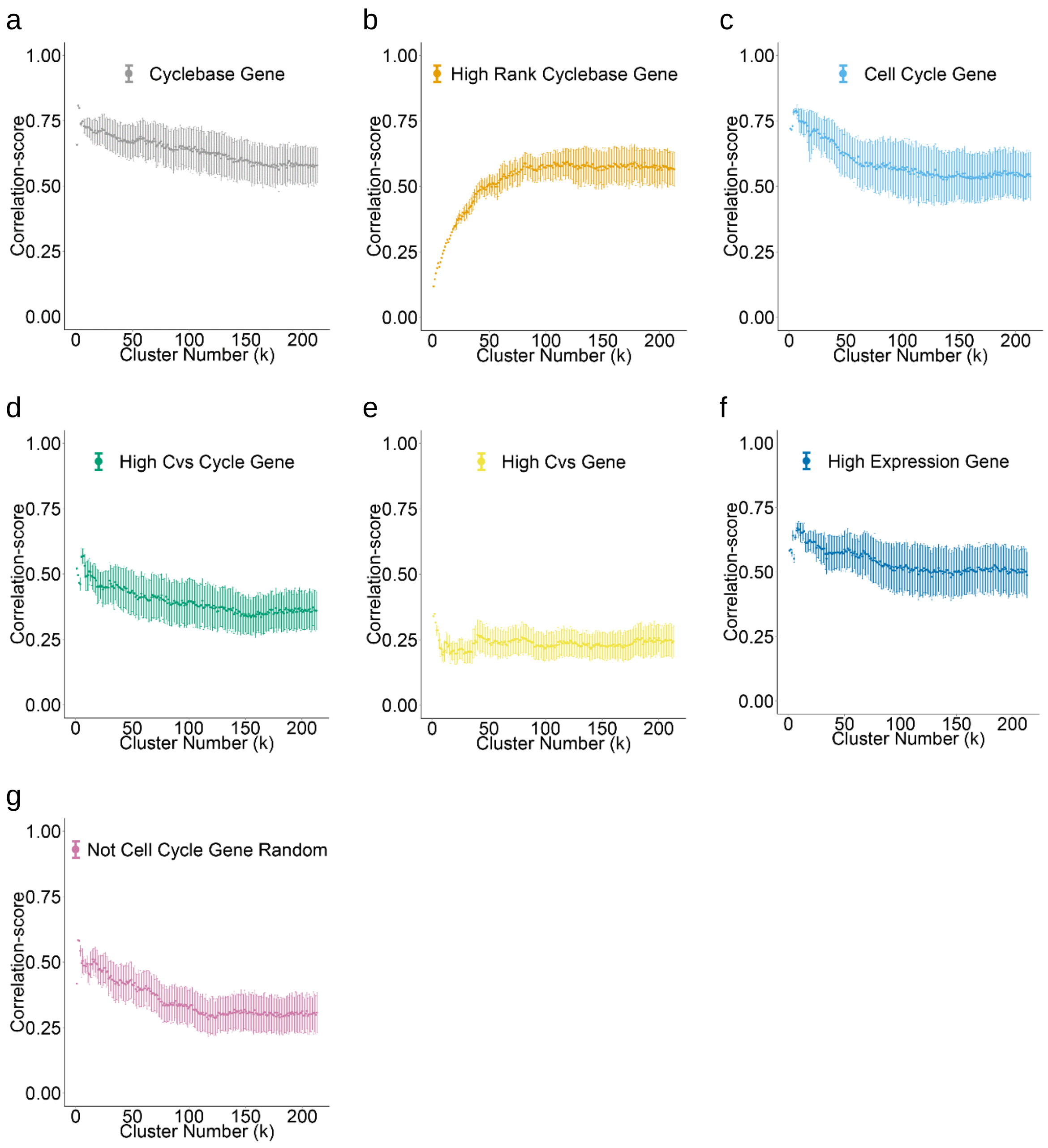

Supplementary Figure 6 | Comparison of seven gene sets for cell cycle analysis using the mESC-SMARTer dataset. A half width of the error bars stands for the standard deviation. (**a**) All 378 Cyclebase genes. (**b**) 120 high rank Cyclebase genes. For each of the six cell cycle stage labels, ‘G1’, ‘G1/S’, ‘S’, ‘G2’, ‘G2/M’ and ‘M’, we selected top 20 genes with the highest significant ranks, for a total of 120 genes forming a list (Supplementary Table 5). (**c**) 872 expressed genes from a list of 892 cell cycle gene list (Buettner, et al)[1]. (**d**) 436 genes with highest coefficient of variation (CV) values, selected (50%) out of the 872 expressed genes. (**e**) 894 genes with highest CV values. (**f**) 888 genes with highest mean expression levels. (**g**) 892 randomly selected non-cell-cycle genes, i.e. excluding the Buettner’s cell cycle genes. The results show that Cyclebase genes and Buettner’s cell cycle genes had best performance among all.

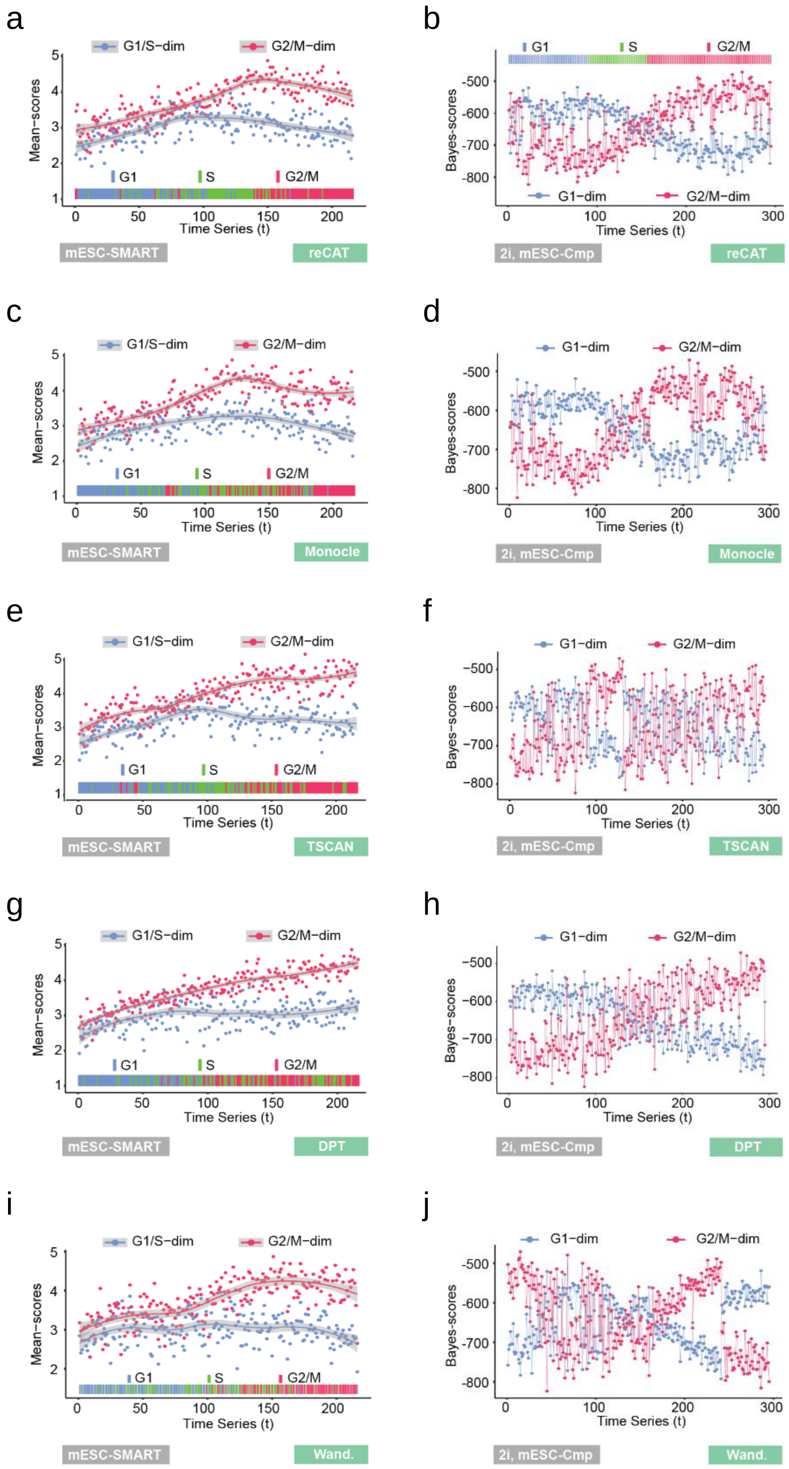

Supplementary Figure 7 | Comparison of reCAT, Monocle, TSCAN, DPT and Wanderlust/Wishbone on stage labeled and unlabeled data sets. Mean-scores of the labeled mESC-SMARTer data arranged by the time-series generated from reCAT (**a**), Monocle (**c**), TSCAN (**e**), DPT (**g**) and Wanderlust/Wishbone (**i**). Bayes-scores of unlabeled 2i samples in the mESC-Cmp data arranged by the time-series generated from reCAT (**b**), Monocle (**d**), TSCAN (**f**), DPT (**h**) and Wanderlust/Wishbone (**j**).

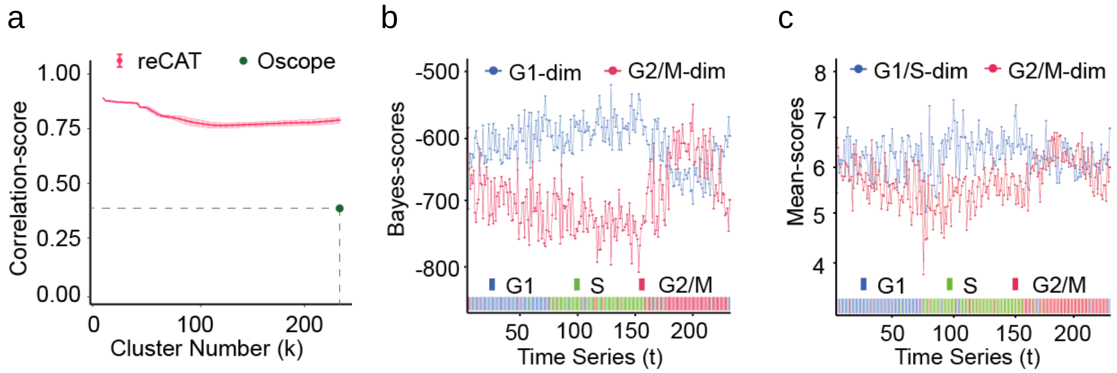

Supplementary Figure 8 | The reCAT recovered cell cycle of the hESC dataset. (**a**) The correlation-score curves for the results by reCAT and Oscope (correlation-score: 0.41), respectively. The Bayes-scores (**b**) and mean-scores (**c**) were plotted with respect to the reCAT-generated time-series at the single cell resolution. The colored bars at the bottom of the panels indicate experimentally determined cell cycle stage labels.

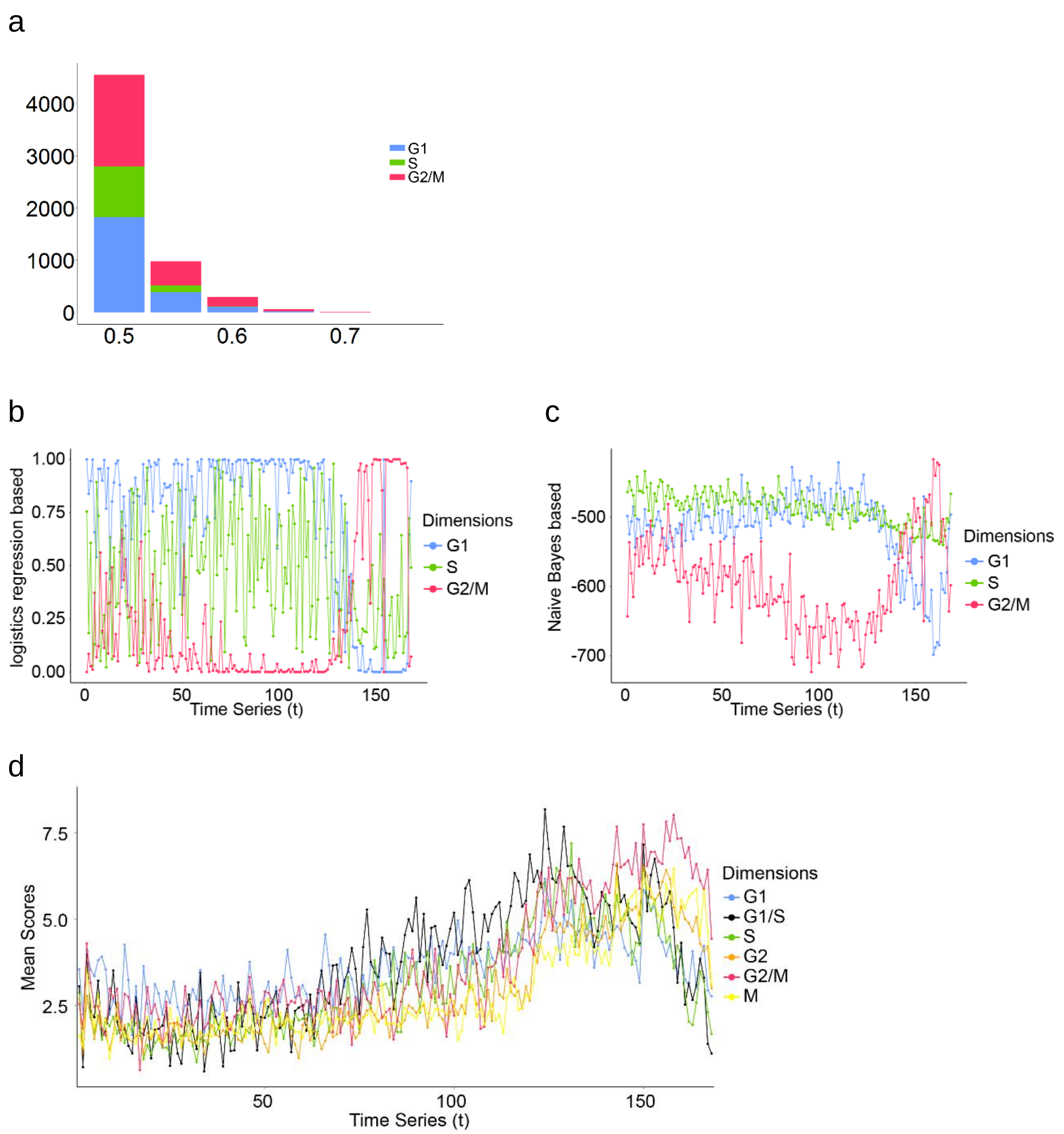

Supplementary Figure 9 | The design of Bayes-scores. (**a**) The numbers of features (y-axis) for cell cycle stages, G1, S, G2/M at each threshold (x-axis). Using the mESC-SMARTer data, we enumerated all pairs of Cyclebase genes, and chose t as a threshold to select eligible pairs as features for each cell cycle stage. Without loss of generality, we focus on the G1 stage and convert the expression of each single cell into a binary vector as follows: For gene *i* and *j,* which form a gene pair, if the proportion of samples with gene expression $e_i > e_j$ is larger than t among G1 samples and the proportion of samples with gene expression $e_i < e_j$ is larger than t among non-G1 samples, then the gene pair was chosen as a feature. Finally, eligible feature pairs of different stages (G1, S, G2M) were unified. The x-axis in the graph stands for the threshold t and the y-axis stands for numbers of the selected feature pairs. Obviously, eligible features are quite few when $t > 0.7$. (**b** - **d**) Comparison among Logistic-Lasso scores (**b**) (Supplementary Methods), Naïve Bayes scores (**c**) and mean-scores (**d**) using the MPP cells from young individuals in the mHSC dataset (young-MPP). The x-axis is the recovered time-series, and the y-axis shows the scores for each cell.

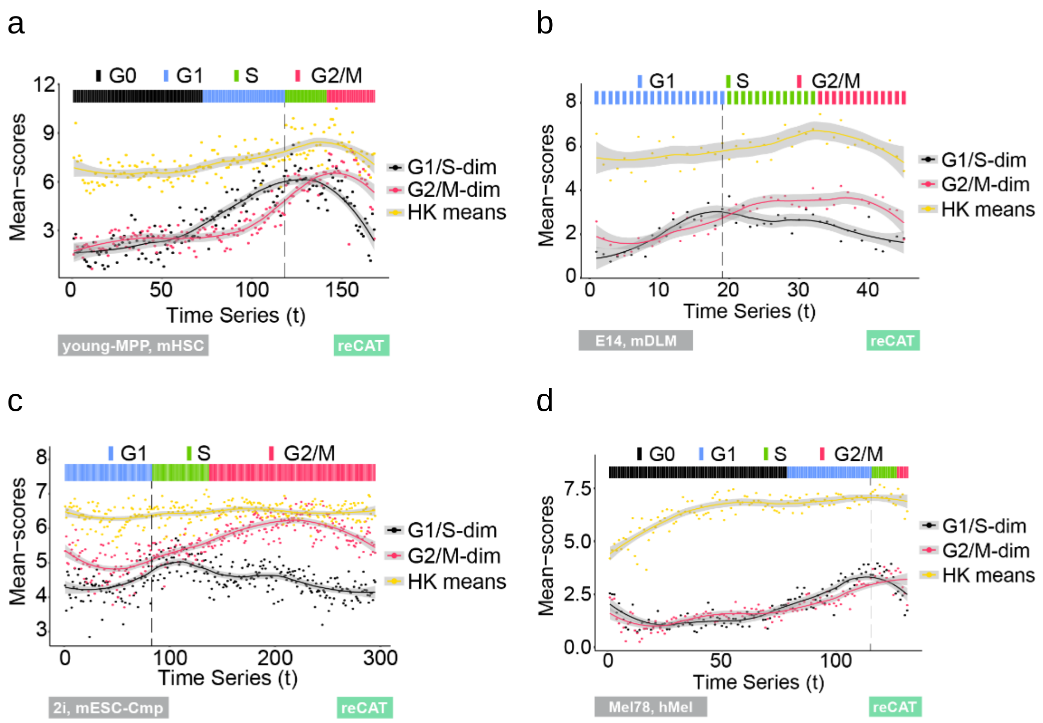

Supplementary Figure 10 | Illustration of mean-scores. Comparisons of G1/S mean-scores, G2/M mean-scores, means of $\log_2$ expression of housekeeping genes (HK) using the young-MPP samples of the mHSC data (**a**), the E14.5 samples of the mDLM data (**b**), the 2i samples of the mESC-Cmp data (**c**) and the Mel78 samples of the hMel data (**d**). The start of the S stage is marked by dashed.

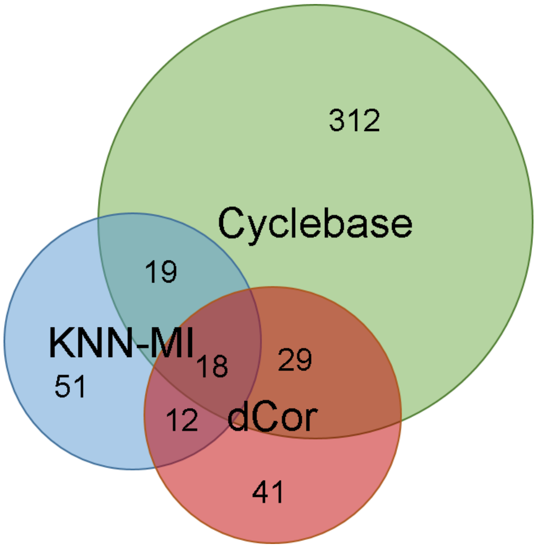

Supplementary Figure 11 | Overlaps among the 378 Cyclebase genes, top 100 genes detected by KNN-MI, and top 100 genes detected by dCor. The latter two gene sets were generated using the young-MPP data from the mHSC dataset, arranged and smoothed along the reCAT-produced time-series.

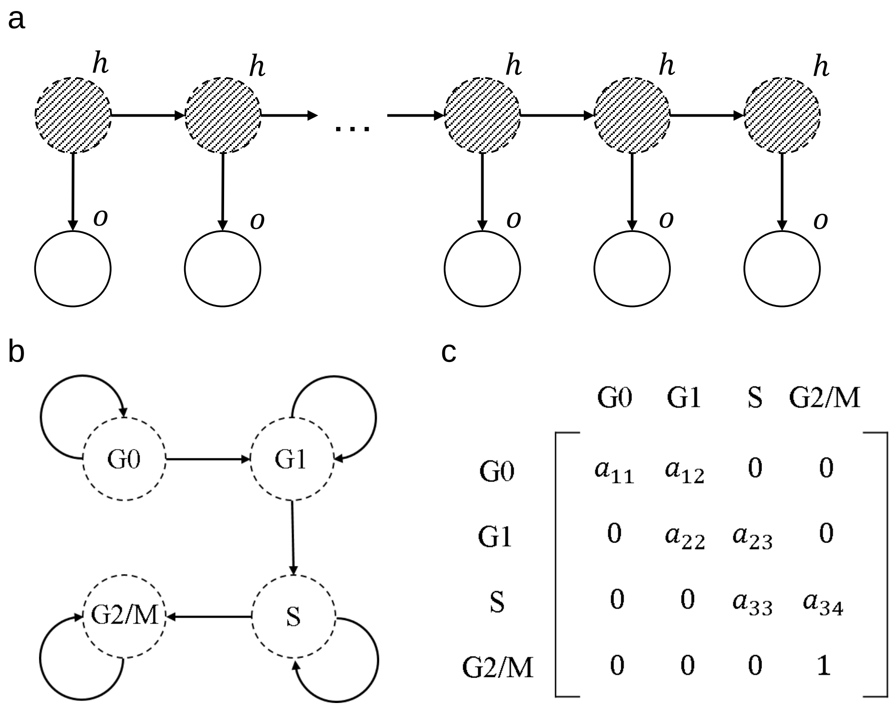

Supplementary Figure 12 | Diagrams for the structure of the HMM model. (**a**) Classical hidden Markov model (HMM) where h_t represents a hidden state, and o_t represents an observation vector. (**b**) The stage transition pattern in our HMM model. (**c**) The matrix of the transition probabilities of the HMM.

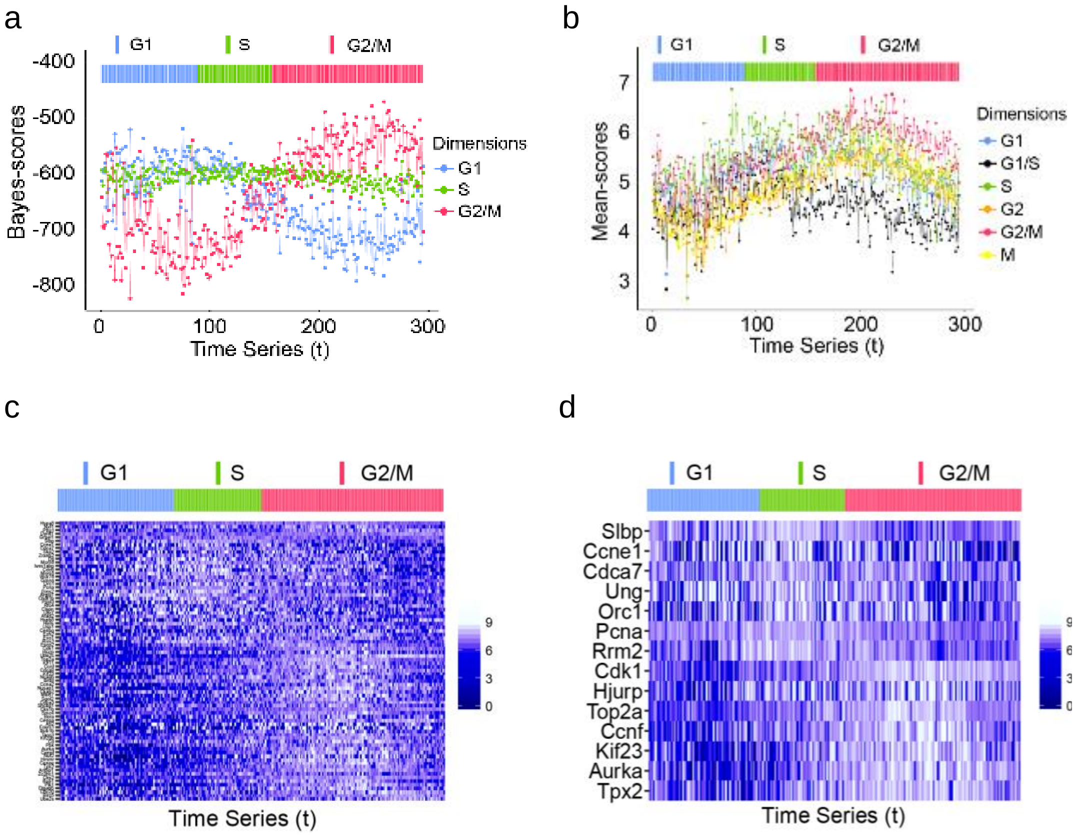

Supplementary Figure 13 | The four panels respectively show the Bayes-scores (**a**) and mean-scores (**b**) for the recovered time-series, time-series expression for the 120 cell cycle stage specific marker genes (Supplementary Table 5) (**c**), and time-series expression levels of 15 high confidence cell cycle marker genes (**d**) of the 2i cells in the mESC-Cmp data set. The heat maps were generated from expression levels without processing by Kalman smoother.

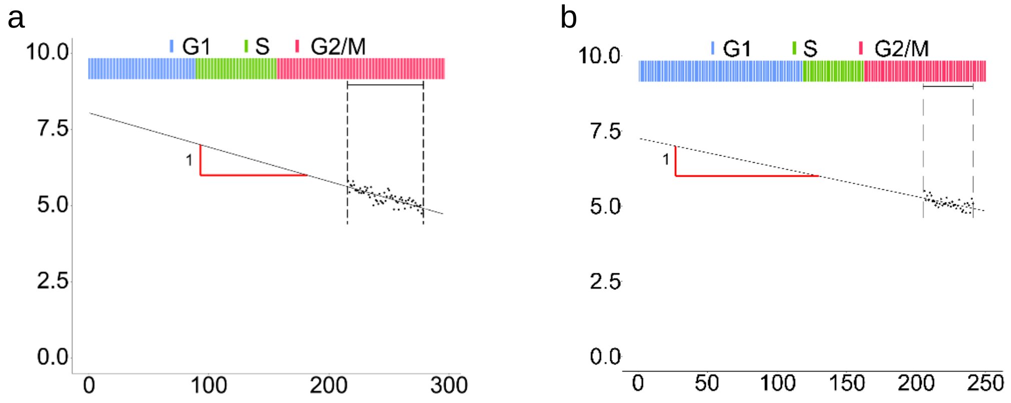

Supplementary Figure 14 | Cell cycle duration estimation leveraging half-time of mRNAs. The samples are 2i cells (**a**) and serum cells (**b**) from the mESC-Cmp data set. The cell cycle time estimation mainly leverage the degradation formula of mRNA, which is also the first order rate equation, $C_t = C_0 e^{-d \cdot t}$. We selected the G2/M cells from the 50% to 90% position (about the second half), in which period most cell cycle related mRNAs are degrading without synthesizing. Then the means of the log2(exp+1) of the Cyclebase genes of were linear regressed by least square regression. The regressed slope $\bar{d}$ is the mean of different degrading rate d. The heights of the vertical lines are 1, thus the horizontal red lines represent the proportion (2i: 88.8/295 = 0.30; Serum: 103.2/250 = 0.41) of the half-time in a cell cycle period. Considering the median of the mRNA half-time of 7h ($\approx \bar{d}$)[2-4], the cell cycle time (doubling time) can be estimated as 7h/0.30 = 23.3h for the 2i samples, and 7h/0.41 = 17.0h for the serum samples. However, according to the original paper of the data, 81 (39 + 42 cells) of the 250 serum samples are reported with much lower proliferation rate than the pluripotency ground state cells (169 cells), then we can propose an approximately equivalent proliferation of 200 (169+31 cells) pluripotency ground state cells (103.2/200 = 0.52) and got a more rational doubling time of 7h/0.52 = 13.5h. This result is very close to the doubling time reported in the original paper (2i: 25h; Serum: 11h).

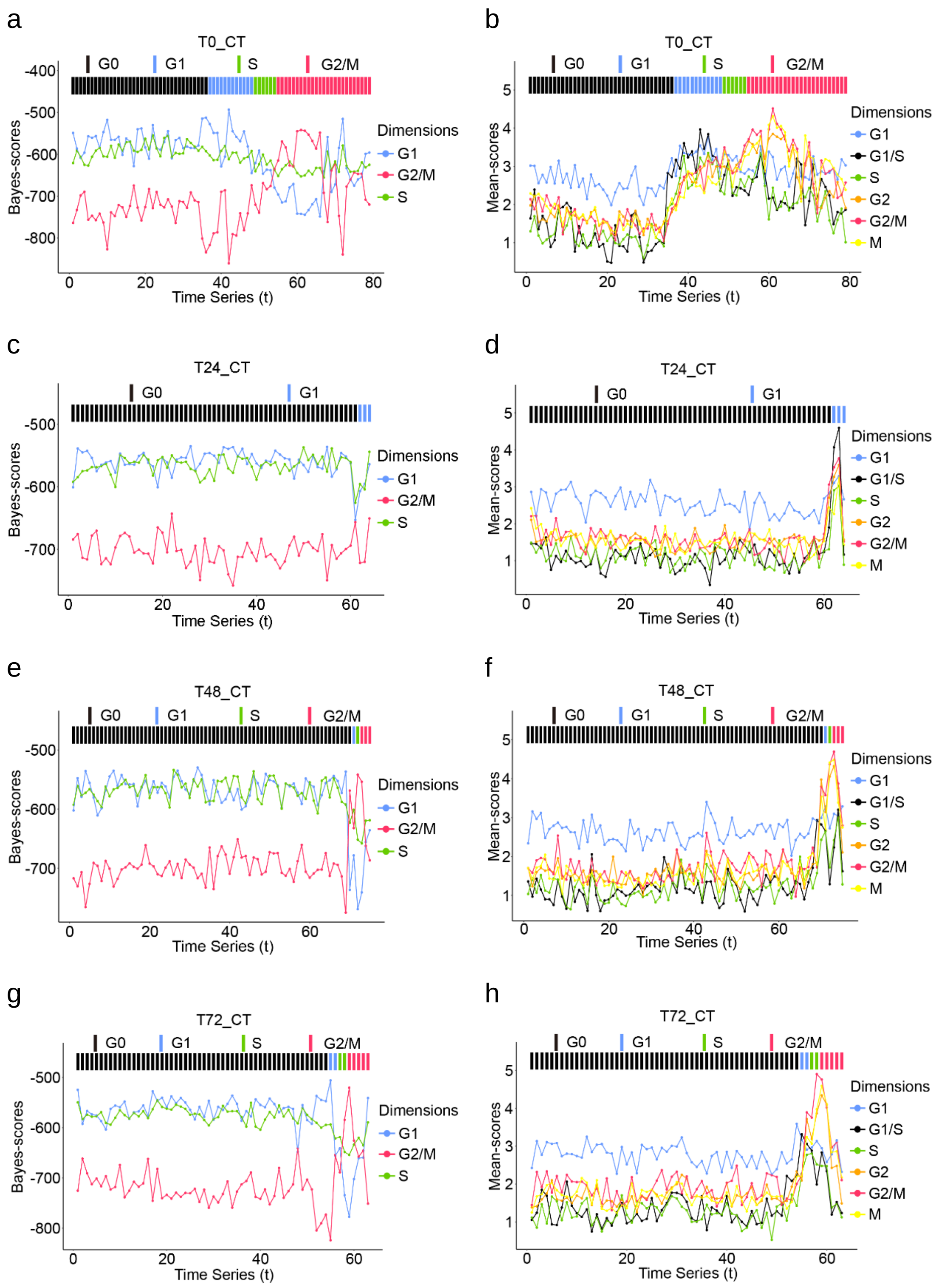

Supplementary Figure 15 | Bayes-scores and mean-scores profiles for cell cycle activities of the hMyo cells (Table 1) at four distinct time points 0th hour (**a**,**b**), 24th hour (**c**,**d**), 48th hour (**e**,**f**), and 72th hour (**g**,**h**) during myoblasts differentiation. After segmentation of cell cycle stages for the T0 cells, the parameters of the HMM were used as initial parameters for segmentations of the other three cell groups.

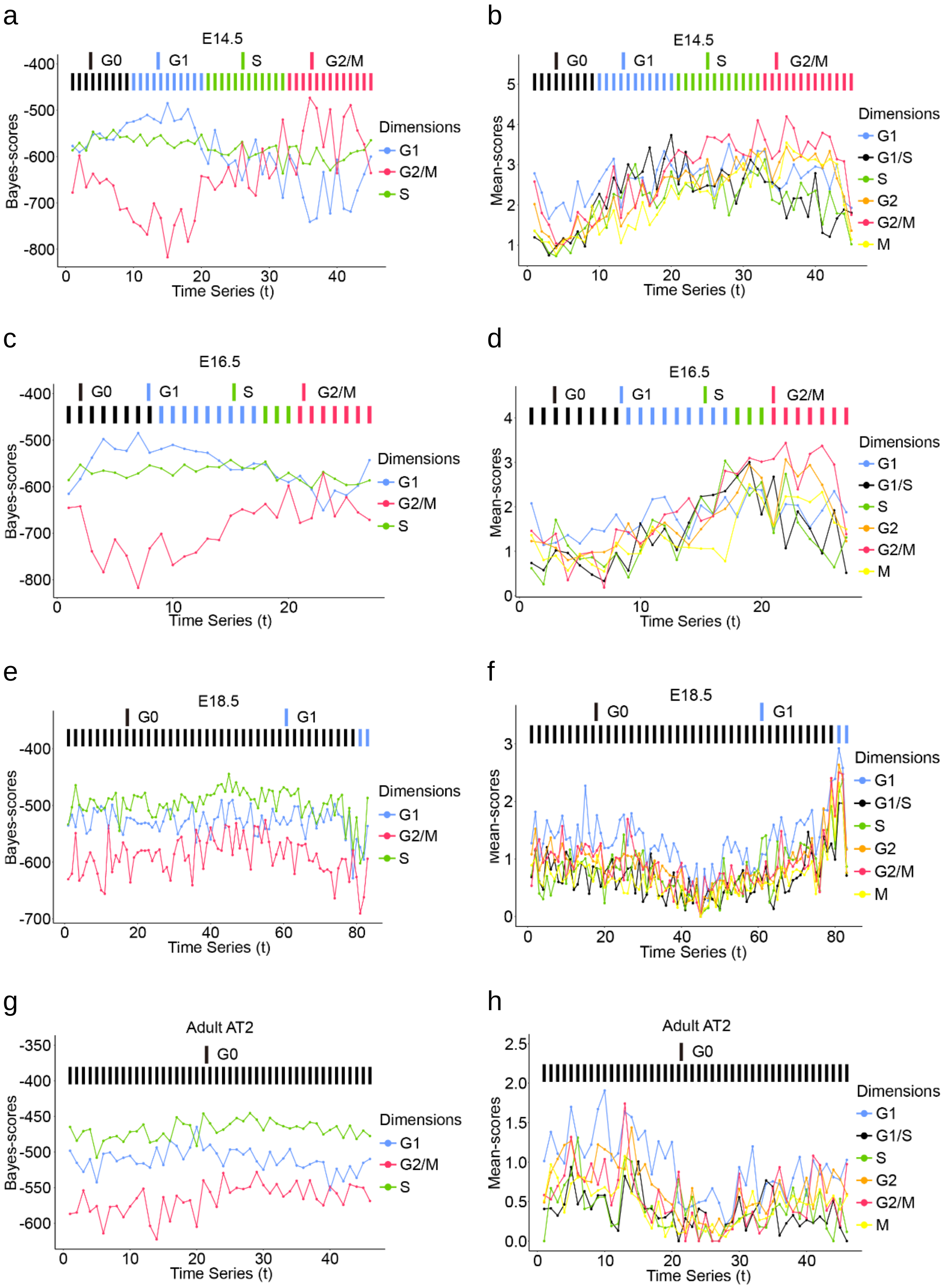

Supplementary Figure 16 | Bayes-scores and mean-scores profiles for cell cycle activities of the mDLM cells (Table 1) at four distinct differentiation stages: E14.5 (**a**,**b**), E16.5 (**c**,**d**), E18.5 (**e**,**f**), and adult AT2 (**g**,**h**) during distal lung epithelium development. After segmentation of cell cycle stages for the E14.5 cells, the parameters of the HMM were used as initial parameters for segmentations of the other three cell groups.

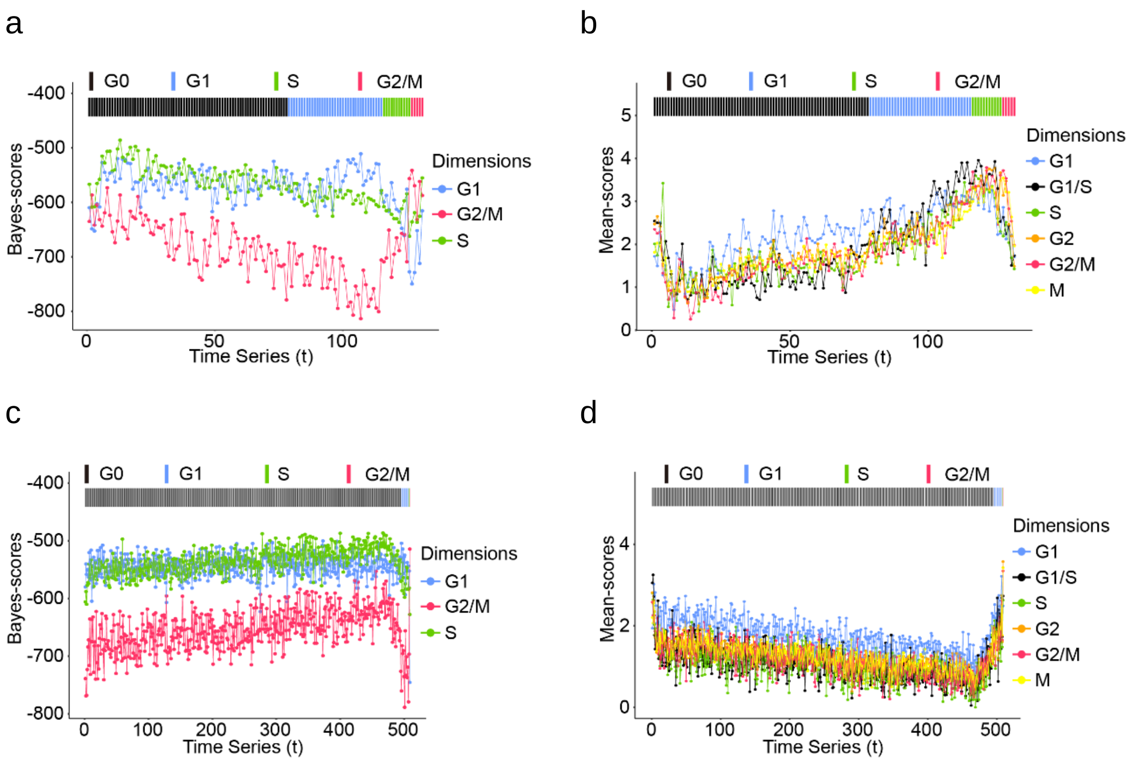

Supplementary Figure 17 | The results of the Mel78 and Mel79 group in hMel dataset (Table 1) processed by reCAT. The color bars on top of each panel indicated the inferred cell cycle stage labels. (**a**, **b**) The Bayes-scores and mean-scores along the pseudo time-series of Mel78 samples. (**c**, **d**) The Bayes-scores and mean-scores along the pseudo time-series of Mel79 samples. Evidently, there is a large fraction of Mel78 cells in cell cycle than that of the Mel79 cells.

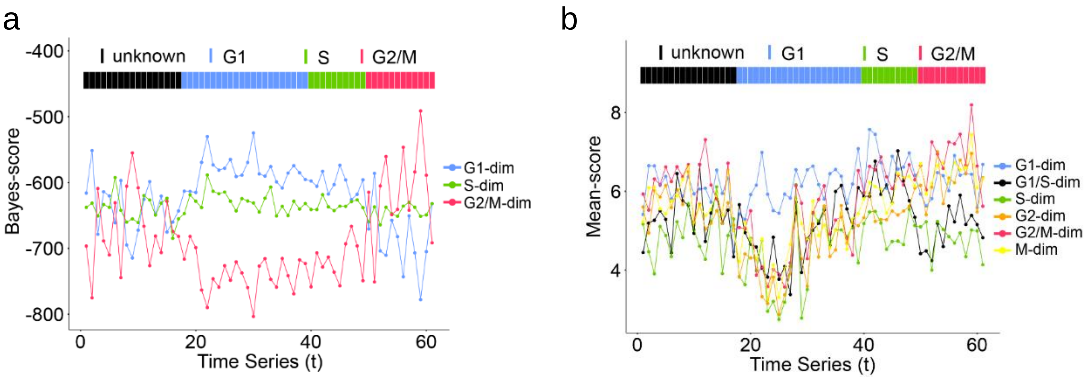

Supplementary Figure 18 | The results of the mESC-MT dataset processed by reCAT. The color bars on top of each panel indicated the cell cycle stage labels inferred by reCAT. (**a**) The Bayes-scores along the pseudo time-series. (**b**) The mean-scores along the pseudo time-series.

Supplementary Table 1 | The top 20 genes for each of the cell cycle stages (G1, S, G2/M) from Cyclebase. (Sorted by 'Peakstage' and 'Rank')

Gene	Source	Identifier	Peakstage	Rank	Peaktime
DTL	Homo sapiens	ENSP00000355958	G1	13	42
CCNE1	Homo sapiens	ENSP00000262643	G1	36	35
PTTG1	Homo sapiens	ENSP00000344936	G1	37	1
CDKN3	Homo sapiens	ENSP00000335357	G1	41	0
ZNF367	Homo sapiens	ENSP00000364405	G1	48	39
SLBP	Homo sapiens	ENSP00000417686	G1	87	34
MCM6	Homo sapiens	ENSP00000264156	G1	114	44
HSPA8	Homo sapiens	ENSP00000227378	G1	115	0
CDCA7	Homo sapiens	ENSP00000306968	G1	148	36
SKP2	Homo sapiens	ENSP00000274255	G1	151	39
ANTXR1	Homo sapiens	ENSP00000301945	G1	154	3
IVNS1ABP	Homo sapiens	ENSP00000356468	G1	159	44
DYNLL1	Homo sapiens	ENSP00000242577	G1	172	4
GRPEL1	Homo sapiens	ENSP00000264954	G1	173	9
ZRANB2	Homo sapiens	ENSP00000359958	G1	174	40
OPN3	Homo sapiens	ENSP00000355512	G1	187	8
KMO	Homo sapiens	ENSP00000355517	G1	188	8
MSL1	Homo sapiens	ENSP00000462945	G1	196	0
NXF1	Homo sapiens	ENSP00000294172	G1	201	0
AOC2	Homo sapiens	ENSP00000253799	G1	210	7
RRM2	Homo sapiens	ENSP00000353770	S	52	64
E2F8	Homo sapiens	ENSP00000250024	S	54	58
ATAD2	Homo sapiens	ENSP00000287394	S	75	55
HSPB8	Homo sapiens	ENSP00000281938	S	116	55
USP1	Homo sapiens	ENSP00000343526	S	129	56
DDX11	Homo sapiens	ENSP00000384703	S	137	54
LIPH	Homo sapiens	ENSP00000296252	S	160	56
DHFRL1	Homo sapiens	ENSP00000319170	S	198	58
HIST2H4B	Homo sapiens	ENSP00000358153	S	206	54
HIST2H4A	Homo sapiens	ENSP00000358162	S	207	54
EZH2	Homo sapiens	ENSP00000320147	S	214	60
CENPQ	Homo sapiens	ENSP00000337289	S	220	56
HELLS	Homo sapiens	ENSP00000239027	S	221	55
EXO1	Homo sapiens	ENSP00000311873	S	235	60
HIST1H4B	Homo sapiens	ENSP00000366581	S	254	62
RHPN1	Homo sapiens	ENSP00000289013	S	261	57
CDKN2AIP	Homo sapiens	ENSP00000427108	S	269	54
EFHC1	Homo sapiens	ENSP00000360107	S	279	56
ASIP	Homo sapiens	ENSP00000364092	S	280	63
WDR90	Homo sapiens	ENSP00000293879	S	283	55

CCNF	Homo sapiens	ENSP00000380256	G2	2	82
CDCA8	Homo sapiens	ENSP00000316121	G2	5	82
HJURP	Homo sapiens	ENSP00000414109	G2	6	76
UBE2C	Homo sapiens	ENSP00000348838	G2	8	81
KIF23	Homo sapiens	ENSP00000260363	G2	11	82
TOP2A	Homo sapiens	ENSP00000411532	G2	14	81
CDK1	Homo sapiens	ENSP00000378699	G2	17	75
CCNA2	Homo sapiens	ENSP00000274026	G2	18	84
NUSAP1	Homo sapiens	ENSP00000453403	G2	26	84
FAM72Bp	Homo sapiens	ENSP00000358397	G2	27	81
FAM72A	Homo sapiens	ENSP00000356096	G2	28	81
KIF11	Homo sapiens	ENSP00000260731	G2	30	81
221690_s_at	Homo sapiens	ENSP00000409370	G2	32	82
PIF1	Homo sapiens	ENSP00000268043	G2	35	84
NCAPH	Homo sapiens	ENSP00000240423	G2	44	82
ARHGEF39	Homo sapiens	ENSP00000367638	G2	45	84
CCDC107	Homo sapiens	ENSP00000414964	G2	46	84
ESCO2	Homo sapiens	ENSP00000306999	G2	51	69
BRD8	Homo sapiens	ENSP00000254900	G2	53	83
CDKN2C	Homo sapiens	ENSP00000262662	G2	58	75

Supplementary Table 2 | 15 high confidence cell cycle genes selected according to published literatures. (Sorted by 'Peaktime')

Gene	Source	Identifier	Peakstage	rank	Peaktime
SLBP	Homo sapiens	ENSP00000417686	G1	87	34
CCNE1	Homo sapiens	ENSP00000262643	G1	36	35
CDCA7	Homo sapiens	ENSP00000306968	G1	148	36
UNG	Homo sapiens	ENSP00000242576	G1	234	39
ORC1	Homo sapiens	ENSP00000360621	G1/S	128	48
PCNA	Homo sapiens	ENSP00000368438	G1/S	43	50
E2F8	Homo sapiens	ENSP00000250024	S	54	58
RRM2	Homo sapiens	ENSP00000353770	S	52	64
CDK1	Homo sapiens	ENSP00000378699	G2	17	75
HJURP	Homo sapiens	ENSP00000414109	G2	6	76
TOP2A	Homo sapiens	ENSP00000411532	G2	14	81
CCNF	Homo sapiens	ENSP00000380256	G2	2	82
KIF23	Homo sapiens	ENSP00000260363	G2	11	82
AURKA	Homo sapiens	ENSP00000216911	M	4	90
TPX2	Homo sapiens	ENSP00000300403	M	9	91

Supplementary Table 3 | Genes whose expression levels were strongly associated with the cell cycle pseudo time-series. There are two methods used for testing, dCor and KNN-MI (Methods). The top 100 genes from each method were respectively ranked by their significant values.

dCor Ranked Genes	dCor Value	Cyclebase or Not	KNN-MI Ranked Genes	KNN-MI Value	Cyclebase or not
Ncapd2	0.91	FALSE	*Hmgb2*	0.95	TRUE
Top2a	0.91	TRUE	*Top2a*	0.95	TRUE
Hmgb2	0.91	TRUE	*H2afz*	0.94	FALSE
Tacc3	0.90	TRUE	*Fen1*	0.92	TRUE
Tubb5	0.89	FALSE	*Tubb5*	0.91	FALSE
H2afz	0.89	FALSE	*Tuba1b*	0.90	FALSE
Bub1b	0.88	TRUE	*Tacc3*	0.90	TRUE
Snrpd1	0.88	FALSE	*Sgol1*	0.90	FALSE
SNORD93	0.87	FALSE	*Ncapd2*	0.87	FALSE
Hmgn2	0.87	FALSE	*Stmn1*	0.86	FALSE
Fen1	0.87	TRUE	*Hmgn2*	0.85	FALSE
Hjurp	0.87	TRUE	*Asf1b*	0.85	TRUE
uc009qbm.2,uc012gva.1	0.86	FALSE	*Bub1b*	0.84	TRUE
Nsl1	0.86	FALSE	*Cdkn3*	0.82	TRUE
Stmn1	0.85	FALSE	*Ccnb1*	0.81	TRUE
Cenpm	0.85	FALSE	*Dnmt1*	0.81	FALSE
Smc2	0.85	FALSE	*SNORD93*	0.81	FALSE
Cdca8	0.85	TRUE	*Kif11*	0.80	TRUE
Hmgb1	0.85	FALSE	*Uhrf1*	0.80	FALSE
Bub1	0.84	TRUE	*Fignl1*	0.79	FALSE
Ncaph	0.84	TRUE	*Hist1h4i*	0.78	FALSE
Rfc5	0.84	FALSE	*Spc25*	0.78	FALSE
Pbk	0.84	TRUE	*Rrm1*	0.77	FALSE
Ncapg2	0.84	FALSE	*Cenpm*	0.77	FALSE
Ezh2	0.84	TRUE	*Fam64a*	0.77	TRUE
Cdk1	0.84	TRUE	*Hmgb1*	0.77	FALSE
Nup85	0.84	FALSE	*Ncaph*	0.77	TRUE
Suv39h1	0.83	FALSE	*Smc2*	0.76	FALSE
Spc25	0.83	FALSE	*Atad2*	0.76	TRUE
Casc5	0.83	FALSE	*Nup85*	0.75	FALSE
Psmd14	0.83	FALSE	*Hjurp*	0.75	TRUE
Dek	0.83	FALSE	*Mcm5*	0.75	TRUE
Ran	0.83	TRUE	*Cdk1*	0.75	TRUE
Kpna2	0.83	FALSE	*Snrpd1*	0.75	FALSE
Tmem48	0.83	FALSE	*Mnd1*	0.75	TRUE
Asf1b	0.83	TRUE	*Kif20b*	0.74	TRUE
Kif20a	0.83	FALSE	*Paics*	0.74	FALSE
Kif22	0.82	FALSE	*Chtf18*	0.73	FALSE
Shcbp1	0.82	TRUE	*Racgap1*	0.73	FALSE
Cks1b	0.82	TRUE	*Tcf19*	0.73	FALSE
D17H6S56E-5	0.82	FALSE	*Dscc1*	0.73	TRUE

Tuba1b	0.82	FALSE	*Pbk*	0.73	TRUE
Nusap1	0.82	TRUE	*Cenpn*	0.72	FALSE
Cenpk	0.82	FALSE	*Ncapg2*	0.72	FALSE
Racgap1	0.82	FALSE	*Rfc5*	0.72	FALSE
Fancd2	0.82	TRUE	*Tk1*	0.71	FALSE
Psma6	0.82	FALSE	*Dctpp1*	0.71	TRUE
Dnajc9	0.82	FALSE	*Bub1*	0.71	TRUE
Dnmt1	0.82	FALSE	*Casc5*	0.71	FALSE
Ccnb1	0.82	TRUE	*Mcm6*	0.71	TRUE
BC030867	0.81	FALSE	*Gins2*	0.71	TRUE
Fam64a	0.81	TRUE	*BC055324*	0.71	FALSE
Nucks1	0.81	FALSE	*Dek*	0.71	FALSE
uc009elc.1	0.81	FALSE	*Oip5*	0.70	FALSE
Mad2l1	0.81	TRUE	*Lig1*	0.70	FALSE
Birc5	0.81	TRUE	*uc009qbm.2,uc012gva.1*	0.70	FALSE
Kif11	0.81	TRUE	*Tipin*	0.70	FALSE
Fanci	0.81	TRUE	*Orc6*	0.70	FALSE
Ckap2l	0.81	TRUE	*Cdc6*	0.70	TRUE
Hist1h1b	0.81	FALSE	*Rpa2*	0.70	FALSE
uc008ave.1,uc008avf.1,uc008avg.1,uc008avh.1,uc008avi.1,uc008avj.1	0.81	FALSE	*Cdca8*	0.70	TRUE
Sgol1	0.81	FALSE	*Aunip*	0.70	FALSE
Tpx2	0.81	TRUE	*Hells*	0.70	TRUE
Ssrp1	0.81	FALSE	*Rad54b*	0.70	FALSE
uc009nvv.1	0.81	FALSE	*Tmem48*	0.69	FALSE
Oip5	0.81	FALSE	*Mcm3*	0.69	FALSE
Impdh2	0.81	FALSE	*Nucks1*	0.69	FALSE
uc008wgd.2,uc012drg.1,uc012drh.1	0.80	FALSE	*Kif4*	0.69	FALSE
uc009qeb.1	0.80	FALSE	*Pcna*	0.68	TRUE
Ttk	0.80	TRUE	*Hist1h2ae*	0.68	FALSE
Cdca3	0.80	TRUE	*uc009nvv.1*	0.68	FALSE
Rfc4	0.80	TRUE	*Cenpi*	0.68	FALSE
Ncapd3	0.80	FALSE	*Cks1b*	0.68	TRUE
Ckap5	0.80	TRUE	*Ran*	0.68	TRUE
Mrpl18	0.80	FALSE	*Ptma*	0.68	FALSE
Clspn	0.80	TRUE	*Nup62*	0.68	FALSE
Lsm3	0.80	FALSE	*Cdc45*	0.68	FALSE
Cenpi	0.80	FALSE	*Cdca7*	0.68	TRUE
Rrm1	0.80	FALSE	*Fancd2*	0.68	TRUE
Ndc80	0.80	TRUE	*uc009qeb.1*	0.68	FALSE
Mrpl42	0.80	FALSE	*Cenpk*	0.68	FALSE
Kif20b	0.79	TRUE	*uc009elc.1*	0.67	FALSE

Psmb6	0.79	FALSE	*Cdca3*	0.67	TRUE
Ncapg	0.79	FALSE	*Ttk*	0.67	TRUE
Orc6	0.79	FALSE	*Gapdh*	0.67	FALSE
Fignl1	0.79	FALSE	*Prim1*	0.67	FALSE
Smc4	0.79	TRUE	*Mrpl18*	0.67	FALSE
Cenpn	0.79	FALSE	*Kif20a*	0.67	FALSE
Nuf2	0.79	TRUE	*uc007bgw.1*	0.67	FALSE
BC055324	0.79	FALSE	*Cdca5*	0.67	TRUE
Psmd11	0.79	FALSE	*Cdc25c*	0.67	TRUE
Uhrf1	0.79	FALSE	*Gm20634*	0.67	FALSE
Kif2c	0.79	TRUE	*Mcm4*	0.66	TRUE
Kif4	0.79	FALSE	*Dnajc9*	0.66	FALSE
Cdca2	0.79	TRUE	*Nsl1*	0.66	FALSE
Kif18a	0.79	FALSE	*Ezh2*	0.66	TRUE
Rad51ap1	0.79	TRUE	*Pmf1*	0.66	FALSE
Gins2	0.78	TRUE	*uc007alb.1*	0.66	FALSE
Exosc8	0.78	FALSE	*Cenpe*	0.66	TRUE
Prc1	0.78	TRUE	*Kif22*	0.66	FALSE

Supplementary Table 4 | The literature support for the top five cell cycle related non-Cyclebase genes detected by KNN-MI and dCor respectively.

**Gene name**	**Statistics**	**Evidence**	**Function**
Ncapd2	KNN-MI & dCor	Gene section on NCBI, GO	Its protein product is cell-cycle regulated. It is required for chromatin-induced microtubule stabilization and spindle formation. (from NCBI)
Tubb5	KNN-MI & dCor	GeneCards®, Gene section on NCBI, GO	The depletion of this gene in vivo perturbs the cell cycle of neurogenic progenitors and alters the position of migrating neurons. (Breuss et al. 2012)
H2afz	KNN-MI & dCor	GeneCards®, Gene section on NCBI	Its overexpression promotes cellular proliferation of breast cancer cells. (Svotelis et al. 2010)
Tuba1b	KNN-MI	GeneCards®	Modification of this gene plays a role in multiple cellular functions, ranging from cell motility, cell cycle progression or cell differentiation to intracellular trafficking and signaling (GeneCards®). It is related to cytoskeleton remodeling (Gene Ontology).
Sgol1	KNN-MI	GeneCards®, Gene section on NCBI, GO	It plays a central role in kinetochore assembly and is required for kinetochore targeting of Plk1. (Pouwels, et al.)
Snrpd1	dCor	Not clearly related	RNA binding (Gene Ontology). No clear function recorded.
Snord93	dCor	Not clearly related (RNA gene)	No clear function recorded.

Supplementary Table 5 | The top 20 genes for each of the cell cycle stages (G1, G1/S, S, G2, G2/M, M) from Cyclebase. (Sorted by 'Peakstage' and 'Rank')

Gene	Source	Identifier	Peakstage	Rank	Peaktime
DTL	Homo sapiens	ENSP00000355958	G1	13	42
CCNE1	Homo sapiens	ENSP00000262643	G1	36	35
PTTG1	Homo sapiens	ENSP00000344936	G1	37	1
CDKN3	Homo sapiens	ENSP00000335357	G1	41	0
ZNF367	Homo sapiens	ENSP00000364405	G1	48	39
SLBP	Homo sapiens	ENSP00000417686	G1	87	34
MCM6	Homo sapiens	ENSP00000264156	G1	114	44
HSPA8	Homo sapiens	ENSP00000227378	G1	115	0
CDCA7	Homo sapiens	ENSP00000306968	G1	148	36
SKP2	Homo sapiens	ENSP00000274255	G1	151	39
ANTXR1	Homo sapiens	ENSP00000301945	G1	154	3
IVNS1ABP	Homo sapiens	ENSP00000356468	G1	159	44
DYNLL1	Homo sapiens	ENSP00000242577	G1	172	4
GRPEL1	Homo sapiens	ENSP00000264954	G1	173	9
ZRANB2	Homo sapiens	ENSP00000359958	G1	174	40
OPN3	Homo sapiens	ENSP00000355512	G1	187	8
KMO	Homo sapiens	ENSP00000355517	G1	188	8
MSL1	Homo sapiens	ENSP00000462945	G1	196	0
NXF1	Homo sapiens	ENSP00000294172	G1	201	0
AOC2	Homo sapiens	ENSP00000253799	G1	210	7
CDC6	Homo sapiens	ENSP00000209728	G1/S	40	46
PCNA	Homo sapiens	ENSP00000368438	G1/S	43	50
MCM5	Homo sapiens	ENSP00000216122	G1/S	67	46
CHAF1B	Homo sapiens	ENSP00000315700	G1/S	73	51
FEN1	Homo sapiens	ENSP00000305480	G1/S	83	52
E2F2	Homo sapiens	ENSP00000355249	G1/S	98	46
POLD3	Homo sapiens	ENSP00000263681	G1/S	102	51
RFC4	Homo sapiens	ENSP00000296273	G1/S	105	52
SERPINB4	Homo sapiens	ENSP00000343445	G1/S	117	45
FAM111B	Homo sapiens	ENSP00000341565	G1/S	125	49
ORC1	Homo sapiens	ENSP00000360621	G1/S	128	48
E2F1	Homo sapiens	ENSP00000345571	G1/S	134	46
WDR76	Homo sapiens	ENSP00000263795	G1/S	163	47
CLSPN	Homo sapiens	ENSP00000312995	G1/S	165	52
UBR7	Homo sapiens	ENSP00000013070	G1/S	166	52
MBOAT1	Homo sapiens	ENSP00000324944	G1/S	178	45
GINS2	Homo sapiens	ENSP00000253462	G1/S	184	50
DONSON	Homo sapiens	ENSP00000307143	G1/S	197	52
GMNN	Homo sapiens	ENSP00000230056	G1/S	202	47
ARGLU1	Homo sapiens	ENSP00000383059	G1/S	215	50
RRM2	Homo sapiens	ENSP00000353770	S	52	64
E2F8	Homo sapiens	ENSP00000250024	S	54	58
ATAD2	Homo sapiens	ENSP00000287394	S	75	55

HSPB8	Homo sapiens	ENSP00000281938	S	116	55
USP1	Homo sapiens	ENSP00000343526	S	129	56
DDX11	Homo sapiens	ENSP00000384703	S	137	54
LIPH	Homo sapiens	ENSP00000296252	S	160	56
DHFRL1	Homo sapiens	ENSP00000319170	S	198	58
HIST2H4B	Homo sapiens	ENSP00000358153	S	206	54
HIST2H4A	Homo sapiens	ENSP00000358162	S	207	54
EZH2	Homo sapiens	ENSP00000320147	S	214	60
CENPQ	Homo sapiens	ENSP00000337289	S	220	56
HELLS	Homo sapiens	ENSP00000239027	S	221	55
EXO1	Homo sapiens	ENSP00000311873	S	235	60
HIST1H4B	Homo sapiens	ENSP00000366581	S	254	62
RHPN1	Homo sapiens	ENSP00000289013	S	261	57
CDKN2AIP	Homo sapiens	ENSP00000427108	S	269	54
EFHC1	Homo sapiens	ENSP00000360107	S	279	56
ASIP	Homo sapiens	ENSP00000364092	S	280	63
WDR90	Homo sapiens	ENSP00000293879	S	283	55
CCNF	Homo sapiens	ENSP00000380256	G2	2	82
CDCA8	Homo sapiens	ENSP00000316121	G2	5	82
HJURP	Homo sapiens	ENSP00000414109	G2	6	76
UBE2C	Homo sapiens	ENSP00000348838	G2	8	81
KIF23	Homo sapiens	ENSP00000260363	G2	11	82
TOP2A	Homo sapiens	ENSP00000411532	G2	14	81
CDK1	Homo sapiens	ENSP00000378699	G2	17	75
CCNA2	Homo sapiens	ENSP00000274026	G2	18	84
NUSAP1	Homo sapiens	ENSP00000453403	G2	26	84
FAM72Bp	Homo sapiens	ENSP00000358397	G2	27	81
FAM72A	Homo sapiens	ENSP00000356096	G2	28	81
KIF11	Homo sapiens	ENSP00000260731	G2	30	81
221690_s_at	Homo sapiens	ENSP00000409370	G2	32	82
PIF1	Homo sapiens	ENSP00000268043	G2	35	84
NCAPH	Homo sapiens	ENSP00000240423	G2	44	82
ARHGEF39	Homo sapiens	ENSP00000367638	G2	45	84
CCDC107	Homo sapiens	ENSP00000414964	G2	46	84
ESCO2	Homo sapiens	ENSP00000306999	G2	51	69
BRD8	Homo sapiens	ENSP00000254900	G2	53	83
CDKN2C	Homo sapiens	ENSP00000262662	G2	58	75
KPNA2p	Homo sapiens	ENSP00000332455	G2/M	10	85
BUB1B	Homo sapiens	ENSP00000287598	G2/M	33	87
TACC3	Homo sapiens	ENSP00000326550	G2/M	34	87
KIF20B	Homo sapiens	ENSP00000260753	G2/M	39	88
CKAP2	Homo sapiens	ENSP00000367276	G2/M	50	85
BORA	Homo sapiens	ENSP00000375082	G2/M	56	86
MKI67	Homo sapiens	ENSP00000357643	G2/M	61	85
EIF5A2	Homo sapiens	ENSP00000295822	G2/M	62	87
CIT	Homo sapiens	ENSP00000376306	G2/M	66	88

CENPA	Homo sapiens	ENSP00000336868	G2/M	76	86
SGOL2	Homo sapiens	ENSP00000350447	G2/M	86	85
ATL2	Homo sapiens	ENSP00000368237	G2/M	88	86
FOXM1	Homo sapiens	ENSP00000342307	G2/M	103	86
CDCA3	Homo sapiens	ENSP00000442068	G2/M	118	85
PBK	Homo sapiens	ENSP00000301905	G2/M	121	88
SHCBP1	Homo sapiens	ENSP00000306473	G2/M	123	85
TTC38	Homo sapiens	ENSP00000370419	G2/M	126	85
BTNL9	Homo sapiens	ENSP00000330200	G2/M	138	85
CKS1B	Homo sapiens	ENSP00000311083	G2/M	139	85
SMC4	Homo sapiens	ENSP00000341382	G2/M	142	85
PLK1	Homo sapiens	ENSP00000300093	M	1	93
MAPK13	Homo sapiens	ENSP00000211287	M	3	93
AURKA	Homo sapiens	ENSP00000216911	M	4	90
CENPE	Homo sapiens	ENSP00000265148	M	7	91
TPX2	Homo sapiens	ENSP00000300403	M	9	91
CKS2	Homo sapiens	ENSP00000364976	M	12	92
BUB1	Homo sapiens	ENSP00000302530	M	15	92
ARL6IP1	Homo sapiens	ENSP00000306788	M	16	91
CENPF	Homo sapiens	ENSP00000355922	M	19	90
DLGAP5	Homo sapiens	ENSP00000247191	M	20	94
UBE2S	Homo sapiens	ENSP00000264552	M	21	96
NUF2	Homo sapiens	ENSP00000271452	M	22	90
HMMR	Homo sapiens	ENSP00000377492	M	23	90
CDC20	Homo sapiens	ENSP00000308450	M	24	94
ZC3HC1	Homo sapiens	ENSP00000351052	M	25	91
LRRC17	Homo sapiens	ENSP00000344242	M	29	92
FAM64A	Homo sapiens	ENSP00000250056	M	31	92
BIRC5	Homo sapiens	ENSP00000301633	M	38	94
DEPDC1B	Homo sapiens	ENSP00000265036	M	42	90
SAPCD2	Homo sapiens	ENSP00000386348	M	47	96

Supplementary Notes

Supplementary Note 1: State-of-the-art in single-cell RNA-seq

Hundreds of thousands of single cells in multiple tissues, including embryonic tissues[5], cancer tissue[6-8], immune[9], neuro[10], and complex tissue[11], have been assayed by scRNA-seq. It was also combined with simultaneous DNA sequencing[12] and methylation sequencing[13]. Several computational methods have been developed for data analysis, including normalization[14], differential expression detection[15], differentiation cascade construction[16], removal of confounding factors[1], oscillatory gene identification[17], transcription dynamics modeling[18] and cell classification[19]. However, cell cycle is not taken into account in most single-cell differentiation studies[20], even though cell cycle activities impact physiological function of cells in so many ways[21].

Supplementary Note 2: Comparisons and selection of computational methods

Selection of TSP methods, and feature gene sets. Among TSP solutions generated by the arbitrary insertion algorithm, the cycle lengths show obvious variation when the cluster number is above 20. The lengths also have apparent negative correlation with the correlation-scores (Supplementary Figure 3c), proving the principle of our approach. It is theoretically impossible to solve a TSP when the cluster number is large, because it is an NP-hard problem. For the different existing heuristic methods[22], when we tested the implementations in the R package 'TSP', nearest insertion show high accuracy and stability (Supplementary Figure 5).When clustering numbers are small, correlation-scores are generally high (Supplementary Figure 5). This could be attributed to greater noise reduction through clustering and closer proximity of the TSP solution to the global optimal for smaller k. Therefore, besides reducing computational time for solving TSP, clustering improves accuracy of the generated time-series.

For feature gene selection, both Cyclebase (378) and Buettner's (892) cell cycle gene sets gave the best results (Supplementary Figure 6**)**. This shows that these known cell cycle genes are the most informative and that a proper number of cell cycle genes can yield more accurate time-series. Thus we chose the Cyclebase (378) gene set for reCAT.

Comparison between Bayes-scores and Lasso-Logistic based score. Using features generated by gene expression comparisons, we compared Naïve Bayes-based Bayes-scores with Lasso-Logistic regression scores (LLR, Supplementary Methods; Supplementary Figure 9). The LLR-based computes a probabilistic score for assigning a cell (or a cell group) to a specific cell cycle stage, but the scores show high variation. In contrast, the Naïve Bayes based method generates smoother scores. Therefore, we adopted the Bayes-scores.

Supplementary Note 3: Complementary analysis

Additional notes for the scores and expression profiles along cell cycle. Bayes-scores performs well in distinguishing between G1 and G2/M stages. If G1 scores are higher than G2/M scores, the corresponding cells are likely to be in G0, G1, or S stages. On the other hand, if G2M scores are higher, the cells are very likely to be in G2/M stage. If there is a smooth crossover between G1 and G2/M, this crossover point is often at the end of the S stage. The highest point of G1/S scores is usually near the start of the S stage, and the highest point of G2/M often occurs before cell division[21]. G0 stage has similar Bayes-scores profiles as G1 stage, but the mean-scores are generally lower, with G1 scores a little higher than the other scores. Therefore, we design an HMM to incorporate these scores to determine each of the cell cycle stages (G0, G1, S and G2/M).

Discussion of experimental technologies to generate cell cycle stage labels. Cell cycle stage labels generated from different experimental technologies can have different accuracies. Though the original paper, which revealed FUCCI, used Hoechst to measure the accuracy of FUCCI[23], no literature reported accuracy comparisons between the two technologies. According to the principles of the two technologies, the resolution of FUCCI may be higher than Hoechst sometime. However, because the definition of cell cycle stage is based on DNA replication and division, we tended to believe Hoechst is more fundamental for cell cycle stage determination. Apparently, both of the technologies give incorrect cell labels, and the ones of the mESC-SMARTer data (Supplementary Figure 7a) may skew training of Bayes-scores in our analyses.

Supplementary Note 4: Further assessment for the pseudo time algorithm

We assessed the results using Bayes-scores and mean-scores, which were developed independently against the tested pseudo time-series construction algorithms. Using the cell cycle-labeled mESC-SMARTer dataset, we plotted mean-scores of the time-series produced by reCAT (Supplementary Figure 7a), Monocle (Supplementary Figure 7c), TSCAN (Supplementary Figure 7e), DPT (Supplementary Figure 7g) and Wanderlust (Supplementary Figure 7i), with labeled cell cycle stages at the bottom of each panel. In spite of the color bars at the bottom, we observed that the G2/M mean-scores (red) of TSCAN and DPT did not decrease at the end of the cell cycle, which is not consistent to the degradation biological properties of the mRNAs[2-4] (Supplementary Figure 14). To explain, TSCAN and DPT have more consideration on linear property of gene expression profiles, not curvilinear or cyclic details. We then plotted Bayes-scores of the time-series produced by reCAT (Supplementary Figure 7b), Monocle (Supplementary Figure 7d), TSCAN (Supplementary Figure 7f), DPT (Supplementary Figure 7h) and Wanderlust (Supplementary Figure 7i), using the unlabeled 2i samples in the mESC-Cmp data. reCAT produced very clean curves, making it possible to clearly distinguish each cell cycle stage, while the Bayes-scores of TSCAN, DPT and Wanderlust time-series are noisy or discontinuous. These results show the superior accuracy of the cell cycle pseudo time-series produced by reCAT.

Since the pseudo time algorithm is based on TSP solving, people may care about its robustness. Therefore, we took the following tests to prove that. It is most straightforward to use labeled data sets. Therefore, for each '*K*' on the horizontal axis, 200 trials were respectively implemented. For each trial, correlation-score was calculated to test the robustness, on the mESC-SMARTer data (Figure 2d), the hESC data (Supplementary Figure 8a) and mESC-Quartz data (Figure 3a).

In summary, the algorithm of reCAT is more reliable for cell cycle pseudo-time reconstruction, mainly because of its accuracy and robustness. The good results of reCAT can first be attributed to the fact that it is based on a circular model that brings in more prior information. Second, it merges many routes together to produce a robust result, similar to the Wanderlust approach[24]. Third, it is a nonlinear method able to fit nonlinear properties of data.

Supplementary Note 5: Further assessment for Bayes-scores and means-scores

As discussed at the beginning of the paper, the greatest challenge faced in delineating cell cycle stages is the high level of uncertainty of marker gene expression. Therefore, Bayes-scores and mean-scores were designed to overcome this uncertainty. The genes used by Bayes-scores and mean-scores are well known and with specific biological explanation[25]. Specifically, Bayes-scores is based on the Naïve Bayesian model that combines a well-behaved cell classification feature selection method[26], which is about expression comparisons of thousands of gene pairs. Mean-scores is based on the means of expression levels of tens of marker genes at each cell cycle stage. Methods similar to mean-scores have been used as a reliable[21].

We demonstrated that the Naïve Bayes model was more robust to noise than other computational methods such as the Logistic regression (Supplementary Note 2, Supplementary Figure 9). To evaluate the

robustness of the Bayes-scores, we trained and tested the Bayes-score method on the labeled mESC-SMARTer dataset. Through ten-fold cross-validation, we found that 94.3% of the G1 cells had higher G1 Bayes-scores than the G2/M Bayes-scores, and that 92.9% of the G2/M cells had higher G2/M Bayes-scores than G1 Bayes-scores. The results demonstrate that the Bayes-scores are very accurate in discriminating between G1 and G2/M cells. We then applied the trained Bayes-score method to discriminate G1 and G2/M cells on the labeled hESC and mESC-Quartz dataset. The results showed that the error rates were 18.4% for the G1 cells and 36.8% for the G2/M cells. While all the G1 and G2/M cells in the mESC-Quartz were correctly discriminated. We did not test the model on the S stage because the S stage is a transitive stage, which can easily be confused with the G1 and G2/M stages.

For mean-scores, we compared G1/S scores with G2/M scores of the mESC-SMARTer data, and plotted these two scores on a 2-dimensional space in which a linear classifier (generated by SVM) achieved 88.9% accuracy to discriminate G1 and G2/M cells. We further showed the robustness of means-scores via comparison with housekeeping genes and random selected genes. For the different types of cells, compared with housekeeping genes and the random selected genes, the curves of G1/S and G2/M dimensions exhibit a clear phase gap along the time-series (Supplementary Figure 10).

Though the calculated values of the scoring methods have some noise and undesirable results, which might because of data qualities, gene selections and stochasticity, the HMM in reCAT can combine all Bayes-scores and mean-scores together to segment the pseudo time series into different cell cycle stages. Besides, the segmentation of HMM model has some robustness to noise and only sensitive to the variation trends of the values but not the values themselves.

Supplementary Methods

Information extraction from the covariance. Covariance matrices of each cluster were calculated as $\mathbf{A}_i$, and PCA (principle component analysis) was performed to extract information within each cluster. For PCA, $\mathbf{A} = \mathbf{U} \cdot \mathbf{\Lambda} \cdot \mathbf{U}'$ where $\mathbf{A}$ is the covariance of all samples, and $\mathbf{\Lambda}$ is a symmetric matrix composed of eigenvalues of $\mathbf{A}$. $\mathbf{U}$ can be estimated and columns of it are composed of homologous eigenvectors. For each covariance matrix $\mathbf{A}_i$ corresponding to cluster i, $\mathbf{\Lambda}_i = \mathbf{U}' \cdot \mathbf{A}_i \cdot \mathbf{U}$. The vector which represents $\mathbf{A}_i$ can be obtained through combining the diagonal elements of $\mathbf{\Lambda}_i$, marked as $\mathbf{v}_i$.

Bayes-scores and mean-scores to assess cell cycle. Bayes-scores and mean-scores reveal the membership of a cell (cluster) for a certain cell cycle stage from two different aspects. Bayes-scores are based on comparison of the gene expression pairs in a cell and integrating the results of a certain number of comparison pairs. Mean-scores are based on comparing specific gene expressions in different samples and integrating the expression into one value by averaging.

We propose a Naïve Bayes model to calculate the likelihood that a cell (cluster) belongs to a specific cell cycle stage. The process is similar to classification of a cell via the Bayes decision rule; thus, we refer to the prediction scores obtained in this way as Bayes-score. Formally, let $\mathbf{G} = \{g_1, \dots, g_m\}$ be the set of annotated cell cycle genes, with expression of g_i denoted by e_i. For each pair of genes g_i and g_j with $i < j$, we compare the expression level using a sign function, defined as

$$q_{ij} = \text{sign}(e_i, e_j) = \begin{cases} -1 & e_i < e_j \\ 1 & e_i \geq e_j \end{cases}.$$

There are two steps in the training phase: feature selection and likelihood estimation. We follow the literature[26] to select a set of gene pairs specific to a cell cycle phase. In detail, given a set of n cells with cell cycle stages labeled as $\mathbf{l} = \{l_1, \dots, l_n\}$ and expression data of cell cycle genes for the k-th cell extracted as $\mathbf{e}_k = \{e_{k1}, \dots, e_{km}\}$, for genes g_i and g_j, we calculate two scores $q^{+}{}_{ij} = \sum_{l_k = \hat{l}} q_{ij}(e_{ki}, e_{kj})$ and $q\ {}_{ij} = \sum_{l_k \neq \hat{l}} q_{ij}(e_{ki}, e_{kj})$ for a certain cell cycle stage $\hat{l}$. Gene pairs with $q^{+}{}_{ij} > 0$ and $q\ {}_{ij} < 0$ are then collected to form a set of feature gene pairs specific to the cell cycle stage $\hat{l}$. Hence, we get feature pairs for G1, S and G2/M stages and unify them to get N_{P} feature pairs. Then, given the cell cycle stage label $\hat{l}$, we estimate the distribution of the feature pairs based on the assumption that the features are mutually independent. The results generate a series of estimates $p(s_i = \hat{s}_i | l = \hat{l})$, where $s_i \in \{s_1, s_2 \dots, s_{N_{\text{P}}}\}$, l is a random variable denoting the cell cycle stage, $s \in \{0,1\}$ which indicates if $e_{ki} \geq e_{kj}$ $(s = 1)$ or not $(s = 0)$ for each pair of expression comparison, and $l \in \{\text{G1}, \text{S}, \text{G2/M}\}$.

In the testing (or scoring) step, the probability of a cell in a specific cell cycle stage is

$$p(l = \hat{l} | \mathbf{s} = \hat{\mathbf{s}}) \propto p(l = \hat{l}) \prod_{i=1}^{N_{\text{P}}} p(s_i = \hat{s}_i | l = \hat{l}),$$

where $\mathbf{s} = (s_1, s_2 \dots, s_{N_{\text{P}}})$ is the binary vector of the comparison results for the feature pairs, and $\hat{\mathbf{s}} = (\hat{s}_1, \hat{s}_2 \dots, \hat{s}_{N_{\text{P}}})$ is the realization of $\mathbf{s}$ in the given cell. We consider the prior distribution of cell cycle phases $p(l = \hat{l})$ to be equal for different stages. Therefore, the Bayes-scores is defined as $\log(\prod_{i=1}^{N_{\text{P}}} p(s_i = \hat{s}_i | l = \hat{l}))$. The training dataset is mESC-SMARTer.

Logistic regression compared with Bayes-score. Logistic regression is a classification method which can offer probabilistic measures. Because the dimension number of a feature vector is far larger than the sample number, Lasso is integrated into the logistic regression. Thus, the gene expression comparison result of a

cell i, i.e. $\mathbf{s}_{\boldsymbol{i}} = (s_{i1}, s_{i2} \dots, s_{iN_{\mathrm{P}}})$, is the input, and the generated probability for a cell in a specific cycle stage $\hat{l}$, which stands for the membership of the cell in that stage, is the output. Without loss of generality, we focus on the G1 stage; thus, the formula of the probability can be expressed as

$$\pi(\mathbf{s}_i) = P(l_i = \mathrm{G1}|\mathbf{s}_i', \boldsymbol{\beta}) = \frac{}{+exp^{(-\boldsymbol{\beta}^T \mathbf{s}_i')}},$$

in which $\mathbf{s}_i' = (1, \mathbf{s}_i)^T$ is the input and $\boldsymbol{\beta} = (\beta_0, \beta_1, \dots, \beta_{N_{\mathrm{P}}})^T$ is a set of coefficients that performs linear transformation on the input vectors $\mathbf{s}_i'$.

We use maximum likelihood to estimate the parameter vector $\boldsymbol{\beta}$, and the objective likelihood function to optimize is as follows

$$l(\boldsymbol{\beta}) = -\sum_{i=1}^{n}[(1 - l_i)\boldsymbol{\beta}^T \mathbf{s}_i' + \ln(1 + \exp(-\boldsymbol{\beta}^T \mathbf{s}_i'))] - \lambda \sum_{k=1}^{N_{\mathrm{P}}}|\beta_k|.$$

The parameters can be estimated by some optimization methods for $\breve{\boldsymbol{\beta}} = arg_{\boldsymbol{\beta}}\, max\, l(\boldsymbol{\beta})$. mESC-SMARTer data were used to train for estimating the parameters.

Additional notes for HMM for segmentation. Procedures of parameter estimation and inference already exist[27]. Scaling is necessary in the implementation of the Baum-Welch re-estimation process. Otherwise, forward and backward probabilities are too small to store in a computer since the sequence is too long.

Before segmentation, we choose a group of initial values for the Baum–Welch algorithm. Because of the various data types, limited cell samples, high level of noise and high demand for accuracy, we choose a confidence interval of 4-10 samples for each stage by direct observation (Supplementary Note 3). Then we use maximum likelihood estimation (MLE) to estimate mean and variance of each dimension of the scores. The obtained means and variances are the required initial values.

Supplementary References

1. Buettner, F. et al. Computational analysis of cell-to-cell heterogeneity in single-cell RNA-sequencing data reveals hidden subpopulations of cells. *Nature Biotechnology* **33**, 155-160 (2015).
2. Sharova, L.V. et al. Database for mRNA half-life of 19 977 genes obtained by DNA microarray analysis of pluripotent and differentiating mouse embryonic stem cells. *DNA Res* **16**, 45-58 (2009).
3. Schwanhausser, B. et al. Global quantification of mammalian gene expression control. *Nature* **473**, 337-342 (2011).
4. Eward, K.L., Van Ert, M.N., Thornton, M. & Helmstetter, C.E. Cyclin mRNA stability does not vary during the cell cycle. *Cell Cycle* **3**, 1057-1061 (2004).
5. Yan, L.Y. et al. Single-cell RNA-Seq profiling of human preimplantation embryos and embryonic stem cells. *Nat Struct Mol Biol* **20**, 1131-+ (2013).
6. Patel, A.P. et al. Single-cell RNA-seq highlights intratumoral heterogeneity in primary glioblastoma. *Science* **344**, 1396-1401 (2014).
7. Miyamoto, D.T. et al. RNA-Seq of single prostate CTCs implicates noncanonical Wnt signaling in antiandrogen resistance. *Science* **349**, 1351-1356 (2015).
8. Tirosh, I. et al. Dissecting the multicellular ecosystem of metastatic melanoma by single-cell RNA-seq. *Science* **352**, 189-196 (2016).
9. Shalek, A.K. et al. Single-cell RNA-seq reveals dynamic paracrine control of cellular variation. *Nature* **510**, 363-369 (2014).
10. Pollen, A.A. et al. Low-coverage single-cell mRNA sequencing reveals cellular heterogeneity and activated signaling pathways in developing cerebral cortex. *Nat Biotechnol* **32**, 1053-1058 (2014).
11. Treutlein, B. et al. Reconstructing lineage hierarchies of the distal lung epithelium using single-cell RNA-seq. *Nature* **509**, 371-+ (2014).
12. Macaulay, I.C. et al. G&T-seq: parallel sequencing of single-cell genomes and transcriptomes. *Nat Methods* **12**, 519-522 (2015).
13. Angermueller, C. et al. Parallel single-cell sequencing links transcriptional and epigenetic heterogeneity. *Nat Methods* (2016).
14. Ding, B. et al. Normalization and noise reduction for single cell RNA-seq experiments. *Bioinformatics* **31**, 2225-2227 (2015).
15. Kharchenko, P.V., Silberstein, L. & Scadden, D.T. Bayesian approach to single-cell differential expression analysis. *Nat Methods* **11**, 740-742 (2014).
16. Trapnell, C. et al. The dynamics and regulators of cell fate decisions are revealed by pseudotemporal ordering of single cells. *Nat Biotechnol* **32**, 381-386 (2014).
17. Leng, N. et al. Oscope identifies oscillatory genes in unsynchronized single-cell RNA-seq experiments. *Nature Methods* **12**, 947-950 (2015).
18. Kim, J.K. & Marioni, J.C. Inferring the kinetics of stochastic gene expression from single-cell RNA-sequencing data. *Genome Biol* **14** (2013).
19. Scialdone, A. et al. Computational assignment Of cell-cycle stage from single-cell transcriptome data. *Methods* **85**, 54-61 (2015).
20. Stegle, O., Teichmann, S.A. & Marioni, J.C. Computational and analytical challenges in single-cell transcriptomics. *Nat Rev Genet* **16**, 133-145 (2015).
21. Kowalczyk, M.S. et al. Single-cell RNA-seq reveals changes in cell cycle and differentiation programs upon aging of hematopoietic stem cells. *Genome Res* (2015).
22. Rosenkrantz, D.J., Stearns, R.E. & Philip M. Lewis, I. An Analysis of Several Heuristics for the Traveling Salesman Problem. *SIAM Journal on Computing* **6**, 563-581 (1977).

23. Sakaue-Sawano, A. et al. Visualizing spatiotemporal dynamics of multicellular cell-cycle progression. *Cell* **132**, 487-498 (2008).
24. Bendall, S.C. et al. Single-cell trajectory detection uncovers progression and regulatory coordination in human B cell development. *Cell* **157**, 714-725 (2014).
25. Ashburner, M. et al. Gene ontology: tool for the unification of biology. The Gene Ontology Consortium. *Nat Genet* **25**, 25-29 (2000).
26. Tan, A.C., Naiman, D.Q., Xu, L., Winslow, R.L. & Geman, D. Simple decision rules for classifying human cancers from gene expression profiles. *Bioinformatics* **21**, 3896-3904 (2005).
27. Rabiner, L.R. A tutorial on hidden Markov models and selected applications in speech recognition. *Proceedings of the IEEE* **77**, 257-286 (1989).
